# Supplementary material for: Heterozygous RFX6 protein truncating variants are associated with MODY with reduced penetrance
Source: Nat Commun. 2017 Oct 12;8:888. doi: 10.1038/s41467-017-00895-9 (PMC5638866; doi:10.1038/s41467-017-00895-9)
Supplement: Supplementary file 1 — Supplementary Information [file 41467_2017_895_MOESM1_ESM.pdf]

Supplementary Table 1: Clinical characteristics of the MODY study cohorts

	Non-Finnish European		Finnish European
	Discovery cohort N=38	Replication cohort N=348	Replication cohort, total n=80
Age of diagnosis (years), median (IQR), n	15 (8), n=38	19.5 (15), n=348	26.5 (26), n=80
Duration of diabetes (years), median (IQR), n	2.5 (12), n=38	3 (10), n=348	1.50 (8), n=80
BMI, median (IQR), n	24 (3), n=21	24 (7), n=275	-
Treatment, n/total N (%)			
Non-insulin treated	15/36 (42%)	154/322 (48%)	45/74 (61%)
Insulin treated	21/36 (68%)	168/322 (52%)	29/74 (39%)
Parental history of diabetes, n/total N (%)	38/38 (100%)	210/346 (61%)	49/70 (70%)
Islet autoantibodies (GAD/IA2 negative, n/total N (%)	38/38 (100%)	203/229 (89%)	26/30 (87%)
Significant endogenous insulin at recruitment*, n/total N (%)	23/25(92%)	211/233(90%)	18/22 (82%)

*noninsulin treated or insulin treated with urine/blood random C-peptide >200 pmol/l.

Supplementary Table 2: List of genes that were included in targeted-next generation sequencing panel.

Gene name	Transcript(s) targeted
ABCC8	U63421 and L78208
BLK	NM_001715.2
CEL	NM_001807.3
EIF2AK3	AF110146.1
FOXP3	NM_014009.2
GATA4	NM_002052.3
GATA6	NM_005257.3
GCK	NM_000162.2
GLIS3	NM_001042413.1
HNF1A	NM_000545.3
HNF1B	NM_000458.1
HNF4A	LRG_483
IER3IP1	NM_016097.3
INS	NM_000207.2
IPF1 (PDX1)	NM_000209.1
KCNJ11	NM_000525.3
KLF11	NM_003597.4
LMNA	NM_005572.2
m.3243 region	NC_012920.1
NEUROD1	NM_002500.2
NEUROG3	NM_020999.2
PAX4	NM_006193.2
PPARG	NM_005169.3
PTF1A	NM_178161.2
RFX6	NM_173560.3
SLC19A2	NM_006996.2
SLC2A2 (GLUT2)	NM_000340.1
WFS1	NM_006005.3
ZFP57	NM_001109809.2

Supplementary Table 3: List of *RFX6* protein truncating variants from study cohorts, ExAC, METSIM and Type 2 Diabetes Knowledge Portal.

							No of heterozygous individuals/total individual				
	cDNA	Protein	Genomic location	Coding effect	Exon	Total No of heterozygous individuals in Exac	Non-Finnish European	Finnish European	African	Latino	Others
Discovery cohort	c.875T>G	p.Leu292Ter	Chr6(GRCh37):g.117237380T>G	Nonsense	9	0					
	c.1051A>T	p.Lys351Ter	Chr6(GRCh37):g.117240328A>T	Nonsense	11	0					
Replication cohort - Non-Finnish European	c.1129C>T	p.Arg377Ter	Chr6(GRCh37):g.117240406C>T	Nonsense	11	0					
	c.73C>T	p.Gln25Ter	Chr6(GRCh37):g.117198511C>T	Nonsense	1	0					
ExAC database	c.858+1G>A	p.?	Chr6(GRCh37):g.117237249G>A	splice donor	8	5	5/33285	0/3302	0/5188	0/5728	0/452
	c.876_877delAC	p.His293Leufs	Chr6(GRCh37):g.117237380TAC>T	frameshift	9	18	1/33352	15/3305	0/5191	0/5770	2/454
	c.1327+1_1327+2insT	p.?	Chr6(GRCh37):g.117241618G>GT	splice donor	12	1	1/33353	0/3302	0/5203	0/5755	0/453
	c.1423_1425delCAGinsC	p.Arg476Serfs	Chr6(GRCh37):g.117243300CAG>C	frameshift	13	1	1/33259	0/3288	0/5175	0/5742	0/453
	c.1477C>T	p.Gln493Ter	Chr6(GRCh37):g.117244309C>T	Nonsense	14	1	1/33346	0/3303	0/5202	0/5772	0/454
	c.1496G>A	p.Trp499Ter	Chr6(GRCh37):g.117244328G>A	Nonsense	14	1	1/33348	0/3302	0/5202	0/5774	0/454
	c.1513C>T	p.Arg505Ter	Chr6(GRCh37):g.117244345C>T	Nonsense	14	1	1/33347	0/3303	0/5202	0/5775	0/454
	c.1573C>T	p.Arg525Ter	Chr6(GRCh37):g.117245849C>T	Nonsense	15	1	1/33351	0/3306	0/5197	0/5782	0/454
	c.1954C>T	p.Arg652Ter	Chr6(GRCh37):g.117248258C>T	Nonsense	17	2	1/33367	0/3307	1/5202	0/5786	0/454
	c.2043C>G	p.Tyr681Ter	Chr6(GRCh37):g.117248347C>G	Nonsense	17	1	1/33369	0/3307	0/5203	0/5786	0/454
	c.2596C>T	p.Arg866Ter	Chr6(GRCh37):g.117250119C>T	Nonsense	18	1	1/33344	0/3306	0/5198	0/5782	0/453
	c.673-1G>T	p.?	Chr6(GRCh37):g.117232097G>T	splice acceptor	6	1	0/33306	0/3304	1/5085	0/5753	0/450
	c.1023-2A>C	p.?	Chr6(GRCh37):g.117240298A>C	splice acceptor	10	1	0/33257	0/3282	0/5169	1/5725	0/452
Total						35	15/33346	15/3305	2/5202	1/5725	2/454
Frequency							0.045%	0.45%	0.038%	0.017%	0.44%
METSIM Study	c.876_877delAC	p.His293Leufs	Chr6(GRCh37):g.117237380TAC>T	frameshift	9	26		26/7040			
Frequency								0.37%			
							All Ethnicity				
Type 2 diabetes knowledge portal	c.858+1G>A	p.?	Chr6(GRCh37):g.117237249G>A	Splice donor	8	5					
	c.876_877delAC	p.His293Leufs	Chr6(GRCh37):g.117237380TAC>T	frameshift	9	18					
	c.1104_1107delCTAA	p.Asp370Argfs	Chr6(GRCh37):g.117240378TCTAA>T	frameshift	11	0		Type 2 diabetes cases	Controls		
	c.1954C>T	p.Arg652Ter	Chr6(GRCh37):g.117248258C>T	Nonsense	17	2					
	c.2043C>G	p.Tyr681Ter	Chr6(GRCh37):g.117248347C>G	Nonsense	17	1					
Total								12/8373	7/8466		
Frequency								0.14%	0.08%		

Supplementary Table 4: Study cohorts and control cohorts - association analysis

	MODY cohorts, Frequency of *RFX6* PTVs	Control population, Frequency of *RFX6* PTV	Sequencing method of control population	Study variants coverage in control population	Odds ratio (95% CI)	*P*
Non-Finnish European	Discovery cohort (n=2/36), 5.5%	ExAC control population (n=15/33346), 0.045%	whole-exome	No. of individuals had 20x coverage at genomic location where variants in MODY were identified<br>Chr6(GRCh37):g.117198511C>T - 94%<br>Chr6(GRCh37):g.117237380T>G – 100%<br>Chr6(GRCh37):g.117240328A>T – 100%<br>Chr6(GRCh37):g.117240406C>T – 100%	131 (14-595)	1.52E-04
	Replication cohort (n=4/348), 1.15%				26 (6-82)	3.83E-05
	Discovery cohort (n=2/36), 5.5%	gnomAD Exome control population (n=25/55858), 0.045%, includes ExAC population	whole-exome	No. of individuals had 20x coverage at genomic location where variants in MODY were identified<br>Chr6(GRCh37):g.117198511C>T - 94%<br>Chr6(GRCh37):g.117237380T>G – 100%<br>Chr6(GRCh37):g.117240328A>T – 100%<br>Chr6(GRCh37):g.117240406C>T – 100%	131 (14-562)	1.40E-04
	Replication cohort (n=4/348), 1.15%				26 (6-76)	3.03E-05
	Discovery cohort (n=2/36), 5.5%	gnomAD Genome control population (n=2/7508), 0.027%, does not include ExAC population	whole-genome	No. of individuals had 20x coverage at genomic location where variants in Study cohort were identified<br>Chr6(GRCh37):g.117198511C>T - 99%<br>Chr6(GRCh37):g.117237380T>G – 99%<br>Chr6(GRCh37):g.117240328A>T – 98%<br>Chr6(GRCh37):g.117240406C>T – 99%	221 (15-3082)	1.32E-04
	Replication cohort (n=4/348), 1.15%				43 (6-483)	5.29E-05
	Discovery cohort (n=2/36), 5.5%	In-house control cohort 1) patients with any known monogenic aetiology , n=297 and 2) HI or NDM patients with unknown causes, n=283 (total n=0/580)	Same analysis pipeline as study cohort, targeted-next generation sequencing	No. of individuals had 20x coverage at genomic location where variants in study cohort were identified<br>Chr6(GRCh37):g.117198511C>T - 100%<br>Chr6(GRCh37):g.117237380T>G – 100%<br>Chr6(GRCh37):g.117240328A>T – 100%<br>Chr6(GRCh37):g.117240406C>T – 100%	-	3.33E-03
	Replication cohort (n=4/348), 1.15%				-	1.96E-02
	Replication cohort excluding exon 1 variants (n=2/348), 0.057%	ExAC control population (n=15/33346), 0.045%	whole-exome	same as above	13 (1.4-55)	1.00E-02
	Replication cohort excluding exon 1 variants (n=2/348), 0.057%	gnomAD Exome control population (n=25/55858), 0.045%, includes ExAC population	whole-exome	same as above	13 (1.4-52)	1.00E-02
	Replication cohort excluding exon 1 variants (n=2/348), 0.057%	gnomAD Genome control population without Exon 1 variant (n=1/7508), 0.013%, does not include ExAC population	whole-genome	same as above	43 (2-2560)	5.70E-03
Finnish European	Replication cohort (n=6/80), 7.5%	METSIM control population, (n=26/7040), 0.37%	whole-exome	no information on coverage	22 (7-56)	1.20E-06
	Replication cohort (n=6/80), 7.5%	SISu control population, (n=36/10489), 0.34%, includes 823 METSIM participants	whole-exome	no information on coverage	23 (8-59)	6.57E-07
	Replication cohort (n=6/80), 7.5%	gnomAD Genome control population (n=10/1747), 0.57%	whole-genome	No. of individuals had 20x coverage at genomic location where variants in MODY were identified<br>Chr6(GRCh37):g.117237380T>G – 99%	14 (4-44)	3.30E-05

Supplementary Table 5: *RFX6* PTVs in gnomAD. Highlighted variants are only seen in gnomAD and were not present in ExAC. Italic variants did not pass filter in gnomAD.

							No of heterozygous individuals/total individual					
gnomAD Exome	cDNA	Protein	Genomic location	Coding effect	Exon	Total No of heterozygous individuals in gnomAD	Non-Finnish European	Finnish European	African	Latino	Others	South asian
	c.285delC	p.Ser95Argfs	Chr6(GRCh37):g.117199019GC>G	frameshift	2	1	1/55858	0/11150	0/7652	0/16791	0/2743	0/15391
	c.673-1G>T	?	Chr6(GRCh37):g.117232097G>T	splice acceptor	6	1	0/55646	0/11148	1/7644	0/16768	0/2740	0/15387
	c.858+1G>A	?	Chr6(GRCh37):g.117237249G>A	splice donor	8	5	4/55469	0/11105	0/7647	0/16748	1/2730	0/15385
	c.858+2T>C	?	Chr6(GRCh37):g.117237250T>C	splice donor	8	1	1/55433	0/11098	0/7647	0/16749	0/2730	0/15386
	c.878_879delAC	p.His293Leufs	Chr6(GRCh37):g.117237380TAC>T	frameshift	9	46	0/55632	46/11136	0/7647	0/16774	0/2735	0/15389
	c.1023-2A>C	?	Chr6(GRCh37):g.117240298A>C	splice acceptor	10	1	0/55698	0/11133	0/7647	1/16755	0/2726	0/15386
	c.1047delT	p.Phe349Leufs	Chr6(GRCh37):g.117240320AT>A	frameshift	11	1	0/55715	0/11142	0/7649	0/16760	0/2732	1/15389
	c.1047_1057delTGCTAAAAATT	p.Phe349Leufs	Chr6(GRCh37):g.117240321TTTTGCTAAAAA>T	frameshift	11	1	1/55714	0/11143	0/7649	0/16760	0/2734	0/15388
	c.1104_1107delAACT	p.Asp370Argfs	Chr6(GRCh37):g.117240378TCTAA>T	frameshift	11	1	1/55684	0/11143	0/7649	0/16758	0/2733	0/15388
	c.1060G>T	p.Glu354Ter	Chr6(GRCh37):g.117240337G>T	Nonsense	11	1	0/55698	0/11142	0/7648	0/16760	0/2734	1/15388
	c.1129C>T	p.Arg377Ter	Chr6(GRCh37):g.117240406C>T	Nonsense	11	1	0/55640	0/11142	1/7649	0/16761	0/2735	0/15387
	c.1327+2dupT	?	Chr6(GRCh37):g.117241618G>GT	splice donor	12	1	1/55730	0/11144	0/7651	0/16763	0/2738	0/15390
	c.1428_1429delAG	p.Arg476Serfs	Chr6(GRCh37):g.117243300CAG>C	frameshift	13	1	1/55780	0/11142	0/7648	0/16769	0/2738	0/15391
	c.1477C>T	p.Gln493Ter	Chr6(GRCh37):g.117244309C>T	Nonsense	14	1	1/55787	0/11147	0/7650	0/16775	0/2739	0/15390
	c.1496G>A	p.Trp499Ter	Chr6(GRCh37):g.117244328G>A	Nonsense	14	1	1/55799	0/11148	0/7650	0/16778	0/2738	0/15390
	c.1513C>T	p.Arg505Ter	Chr6(GRCh37):g.117244345C>T	Nonsense	14	2	2/55794	0/11147	0/7649	0/16778	0/2738	0/15389
	c.1554_1555insATAATGCACGTATGTTACCAAAACTGGATGCATTGTTCAAGGTGAGATTATGCATTACTCGAGCACCAAAAAAACTCCACTTTAACAATGTATCCAGTTTT	*p.Gly519Ilefs*	*Chr6(GRCh37):g.117244376C>CATCCAGTTTTATAATGCACGTATGTTACCAAAACTGGATGCATTGTTCAAGGTGAGATTATGCATTACTCGAGCACCAAAAAAACTCCACTTTAACAATGT*	*Nonsense*	*14*	*1*	*1/55786*	*0/11145*	*0/7648*	*0/16777*	*0/2737*	*0/15389*
	c.1573C>T	*p.Arg525Ter*	*Chr6(GRCh37):g.117245849C>T*	*Nonsense*	*15*	*1*	*1/55777*	*0/11144*	*0/7648*	*0/16785*	*0/2739*	*0/15391*
	c.1723delC	p.Leu575Trpfs	Chr6(GRCh37):g.117246659TC>T	frameshift	16	1	1/55727	0/11150	0/7651	0/16788	0/2742	0/15391
	c.1724delT	p.Leu575Argfs	Chr6(GRCh37):g.117246660CT>C	frameshift	16	1	1/55729	0/11150	0/7651	0/16788	0/2742	0/15391
	c.2303delA	p.Asn768Ilefs	Chr6(GRCh37):g.117248605CA>C	frameshift	17	1	1/55675	0/11147	0/7641	0/16780	0/2739	0/15390
	c.2399-1G>A	?	Chr6(GRCh37):g.117249921G>A	splice acceptor	17	1	1/55776	0/11147	0/7651	0/16782	0/2740	0/15390
	c.1954C>T	p.Arg652Ter	Chr6(GRCh37):g.117248258C>T	Nonsense	17	4	3/55794	0/11144	1/7650	0/16789	0/2741	0/15389
	c.2043C>G	p.Tyr681Ter	Chr6(GRCh37):g.117248347C>G	Nonsense	17	1	0/55800	0/11150	0/7651	0/16787	.01/2742	0/15391
	c.2176C>T	p.Arg726Ter	Chr6(GRCh37):g.117248480C>T	Nonsense	17	1	0/55661	0/11133	0/7652	0/16783	0/2738	1/15389
	c.2463_2464insA	p.Val822Serfs	Chr6(GRCh37):g.117249986C>CA	frameshift	18	1	1/55837	0/11147	0/7652	0/16787	0/2741	0/15391
	c.2596C>T	p.Arg866Ter	Chr6(GRCh37):g.117250119C>T	Nonsense	18	1	1/55795	0/11149	0/7652	0/16787	0/2740	0/15391
Total						80	25/55858	46/11150	3/7652	1/16791	.2/2742	3/15391
Frequency (%)							0.045	0.413	0.039	0.006	0.073	0.019

gnomAD Genome	cDNA	Protein	Genomic location	Coding effect	Exon	Total No of heterozygous individuals in Exac	Non-Finnish European	Finnish European	African	Latino	Others	South asian
	c.73C>T*	p.Gln25Ter	Chr6(GRCh37):g.117198511C>T	Nonsense	1	1	1/7495	0/1742	0/4357	0/416	0/487	0
	c.878_879delAC*	p.His293LeufsTer7	Chr6(GRCh37):g.117237380TAC>T	frameshift	9	10	0/7493	10/1747	0/4361	0/418	0/491	0
	c.2088_2089delTT	p.Phe696LeufsTer2	Chr6(GRCh37):g.117248389CTT>C	frameshift	17	1	1/7508	0/1747	0/4364	0/419	0/490	0
	c.2596C>T	p.Arg866Ter	Chr6(GRCh37):g.117250119C>T	Nonsense	18	1	0/7504	0/1747	0/4364	1/418	0/490	0
Total						13	2/7508	10/1747	0/4364	1/419	0/490	0
Frequency(%)							0.027	0.57	0	0.24	0	0

Supplementary Fig. 1: Pedigrees of families with heterozygous *RFX6* PTVs who were identified in Non-Finnish replication cohort. Genotype is shown underneath each symbol; M and N denote mutant and wild-type alleles, respectively. Directly below the genotype is the age of diabetes onset in years, duration in years, BMI and treatment at study entry. Squares represent male family members, and circles represent female sex. Black-filled symbols denote patients with diabetes, an arrow denotes the proband in the family. OHA, oral hypoglycaemic agents.

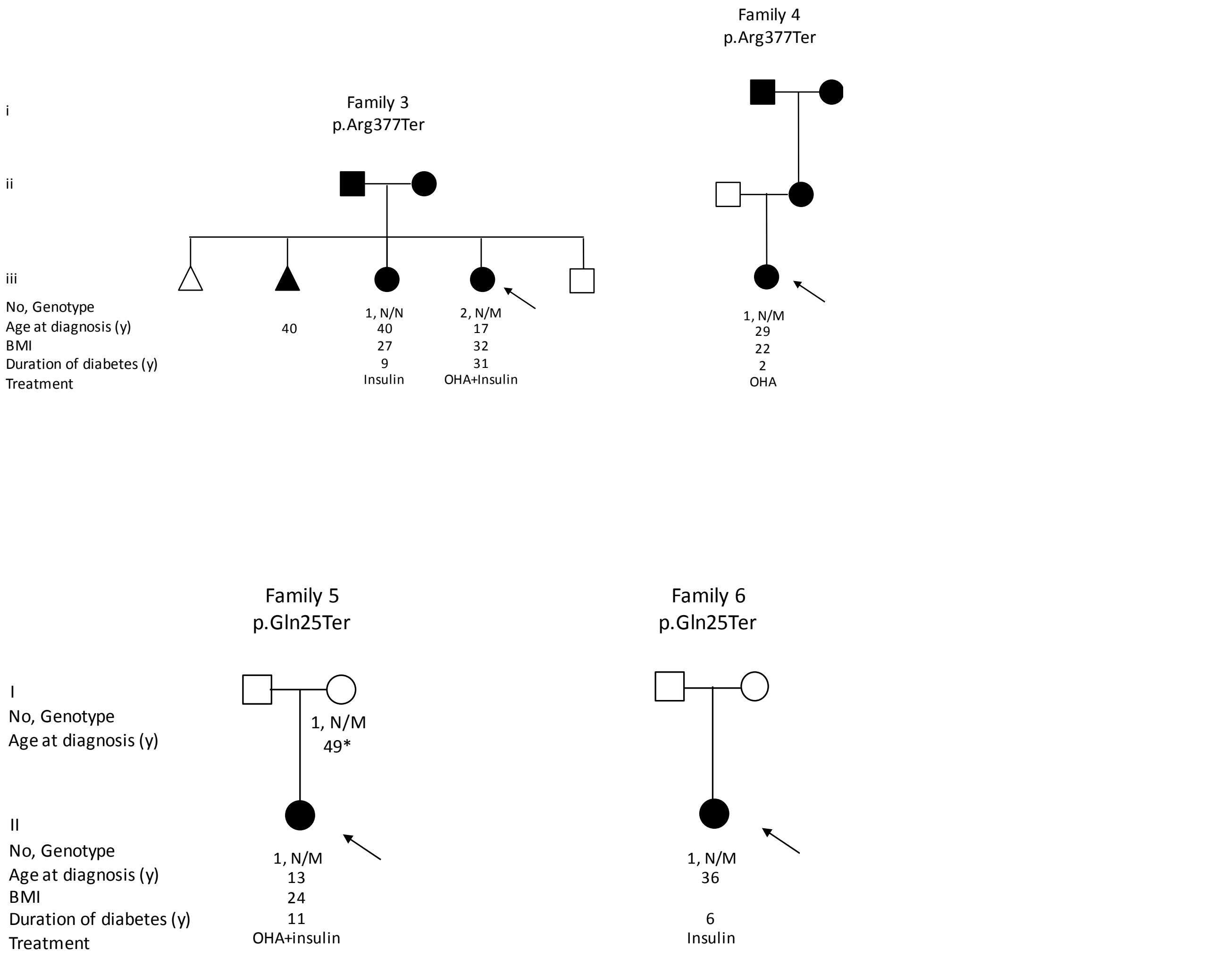

Supplementary Fig. 2: Pedigrees of Finnish families with heterozygous *RFX6* p.His293Leufs variant. Genotype is shown underneath each symbol; M and N denote mutant and wild-type alleles, respectively. Directly below the genotype is the age of diabetes onset in years, duration in years, BMI and treatment at study entry. Squares represent male family members, and circles represent female sex. Black-filled symbol denotes diabetes, gray denotes IGT/IFG, red denotes neonatal diabetes. An arrow denotes the proband in the family. OHA, oral hypoglycaemic agents. Family 1 and Family 2 were part of the replication cohort whereas Family 3, 4 and 5 were identified separately. Family 4 has been previously described by Huopio *et al*.[23]

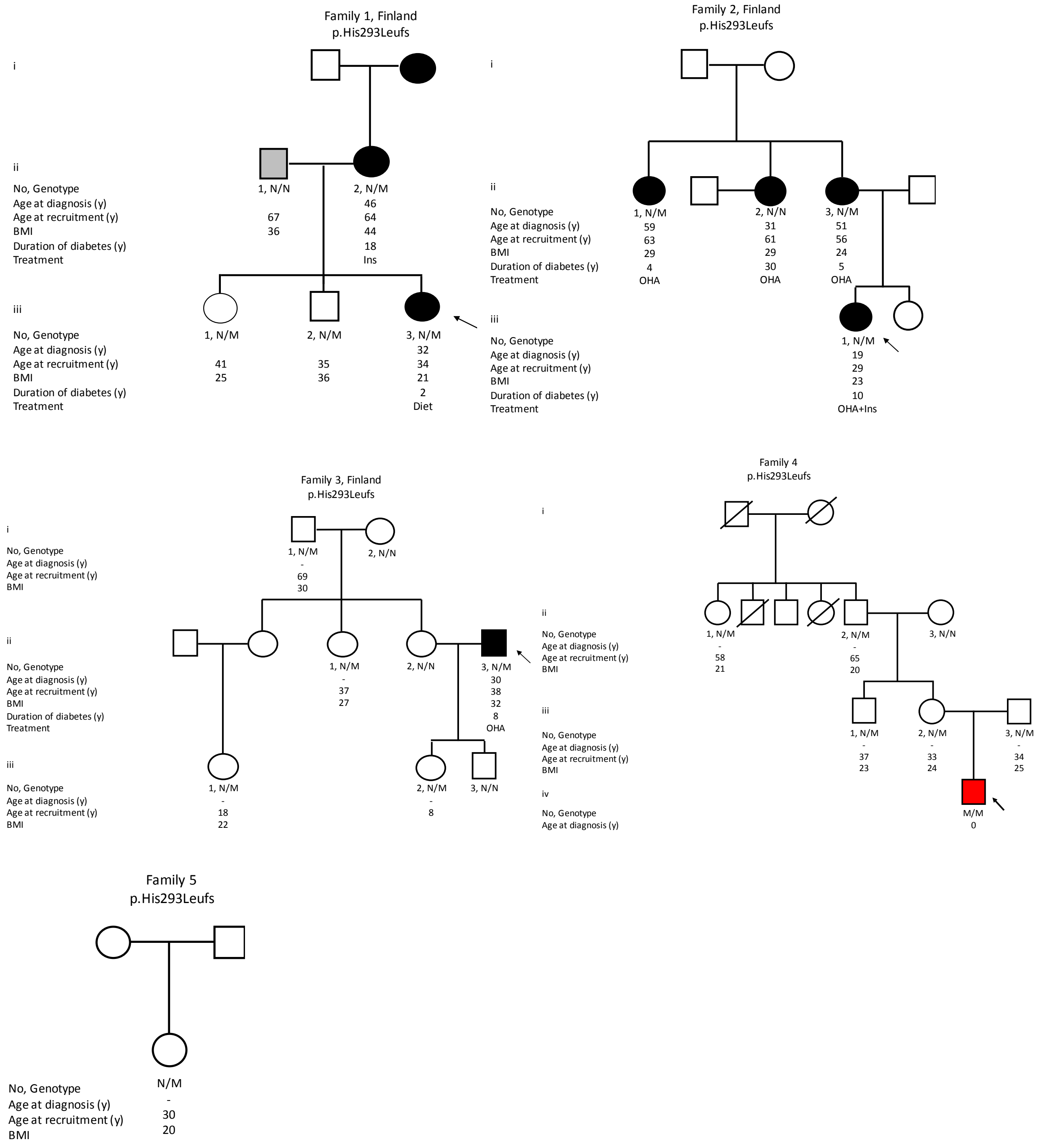

Supplementary Fig. 3: Pedigree of the family with *RFX6* Neonatal diabetes due to homozygous missense variant (p.R181Q/p.R181Q). This family is previously reported by Martinovici *et al*[18] and Smith *et al*[15]. Genotype is shown underneath each symbol; M and N denote mutant and wild-type alleles, respectively. Directly below the genotype is the age of diabetes onset in years, duration in years, age of recruitment, BMI and treatment at study entry. Squares represent male family members, and circles represent female sex. An arrow denotes the proband in the family.

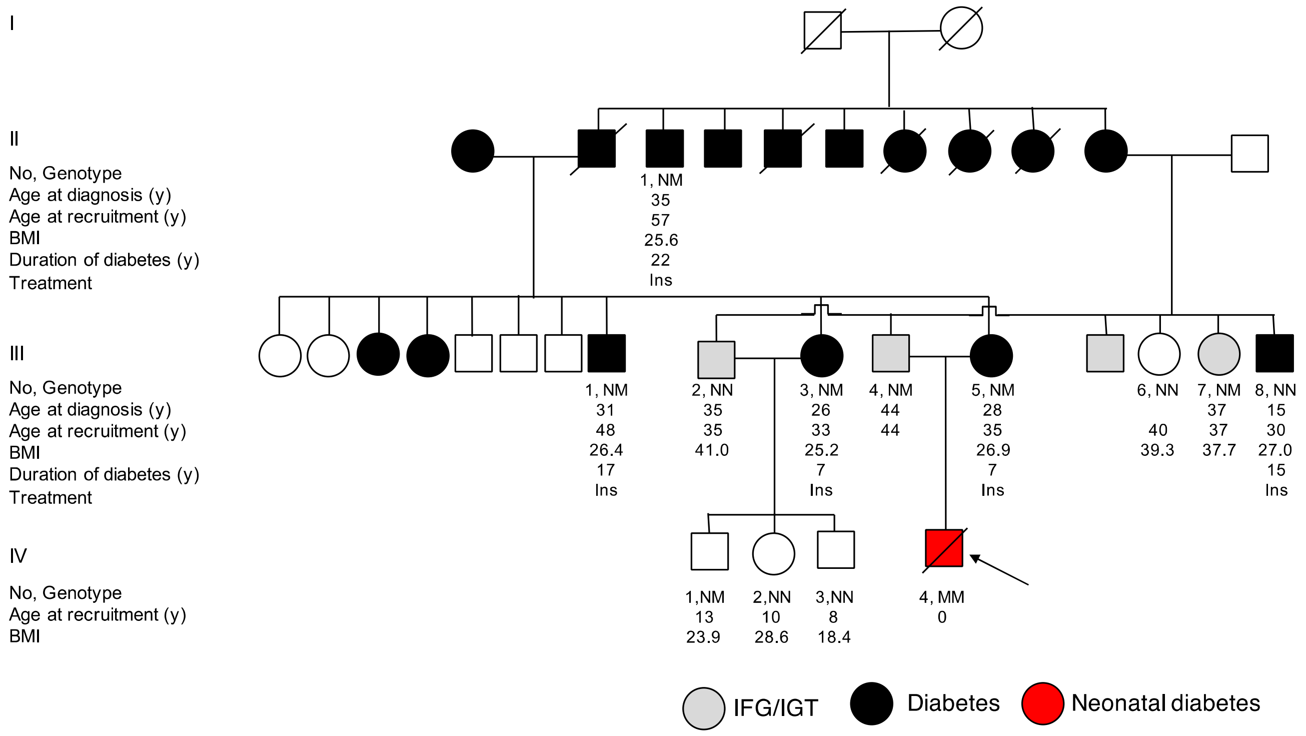

Supplementary Table 6: Phenotypic characteristics of the Finnish *RFX6* p.His293Leufs carriers and population controls from the PPP-Botnia Study (1:5 matched for age, sex and BMI)

		RFX6 p.His293Leufs carriers All (N=16)	Controls N=80	*P*	RFX6 p.His293Leufs carriers No diabetes (N=11)	Controls N=55	*P*
diabetes (N)		5	3	$1.7E^{-03}$	NA	NA	
gender (M/F)		6/10	30/50	1.00	5/6	25/30	1.00
age		37.8 (33.4–59.3)	40.7 (33.1–57.3)	0.82	36.3 (33.1–49.7)	36.5 (32.6–56.3)	0.93
BMI (kg/m^2)		24.4 (22.5–28.9)	24.5 (22.5–28.8)	0.99	24.1 (21.2–26.2)	23.9 (21.5–26.5)	0.90
S-GIP (pg/ml)*	0’	19.2 (11.3–26.9) (n=9/16)	54.3 (32.6–64)	$1.5E^{-04}$	18.3 (9.5–33.5) (n=6/11)	48.9 (29.1–60.2)	$8.6E^{-03}$
	120'	160 (129–215) (n=10/16)	259 (200–328)	$2.7E^{-03}$	167 (134 - 237) (n=8/11)	241 (200–315)	0.029
S-GLP-1 (total)*	0'	10 (9.2–11.1) (n=8/16)	10.8 (9.4–13.8)	0.61	9.7 (9.1–10.1) (n=6/11)	10.6 (9–13.1)	0.58
	120'	23.9 (22.9–25.6) (n=7/16)	18.7 (14.5–23) (71/80)	0.041	24.6 (22.6–25.8) (n=2/11)	18.9 (14.9–22.3) (n=48/55)	0.047
P-glucose (mmol/l)*	0’	5.9 (5.4–6.8)	5.2 (4.8–5.5)	$2.5E^{-04}$	5.5 (5.3–5.9)	5.1 (4.8–5.5)	0.02
	30'	8.9 (8–9.2) (n=14/16)	7.7 (6.9–9)	0.11	8.4 (7.5–9)	7.6 (6.9–9)	0.51
	120'	5.4 (4.5–6) (n=14/16)	4.9 (4.2–5.8)	0.17	5.1 (4.5–5.5)	4.9 (4.1–5.8)	0.73
S-insulin (mU/l)	0’	6.0 (5.2–8.3)	6.0 (4.1–9.1)	0.65	5.7 (5.5–7.8)	5.7 (3.9–8.2)	0.41
	30'	71.5 (34.1–90.4) (n=13/16)	50.2 (34–74.4)	0.40	83.6 (70.8–93.7) (n=10/11)	45.3 (33.5–71.6)	0.015
	120'	28.1 (19.3–43.4) (n=14/16)	22 (15.8–35.4)	0.16	30.1 (20.5–49.8)	21.5 (16.4–32.4)	0.10
S-C-peptide (nmol/l)	0’	0.54 (0.50–0.68)	0.45 (0.31–0.59)	0.014	0.54 (0.51–0.62)	0.41 (0.31–0.55)	$7.5E^{-03}$
	120'	1.96 (1.66–2.27) (n=14/16)	1.34 (1.00–1.76)	$5.8E^{-03}$	1.89 (1.69–2.20)	1.25 (0.95–1.59)	$1.2E^{-03}$
HOMA-IR		1.57 (1.32–2.38)	1.31 (0.92–2.15)	0.053	1.47 (1.28–1.91)	1.30 (0.92–1.92)	0.17
CIR		201 (153–251) (n=13/16)	170 (84–357)	0.82	236 (201–279) (n=10/11)	160 (87–318)	0.09
ISI** (1000/(mmol/l*mU/l))		10.8 (8.4–13.6) (n=13/16)	15.0 (10.2–20.1)	0.04	10.8 (9.0–12.3) (n=10/11)	15.6 (11.6–20.3)	0.018
S-cholesterol (mmol/l)		4.9 (4.4–5.5)	5.2 (4.3–6.1)	0.69	4.9 (4.4–5.4)	5.1 (4.2–5.8)	0.89
S-HDL cholesterol (mmol/l)		1.67 (1.18–1.93)	1.44 (1.08–1.72)	0.31	1.66 (1.10–1.82)	1.44 (1.22–1.77)	0.84
S-LDL cholesterol (mmol/l)		3.1 (2.7–3.5) (n=14/16)	3.0 (2.4–4.0)	0.82	3.1 (2.9–3.5)	3.0 (2.2–3.9)	0.83
S-triglycerides (mmol/l)		0.92 (0.70–1.32)	0.99 (0.73–1.42)	0.40	0.86 (0.70–1.13)	0.95 (0.67–1.21)	0.61
systolic blood pressure (mmHg)		124 (111–142) (n=15/16)	128 (116–137)	0.78	124 (110–142)	125 (116–137)	0.70
diastolic blood pressure (mmHg)		77 (68–85) (n=15/16)	72 (68–80)	0.31	77 (65–80)	70 (66–80)	0.72

Data are median (IQR); HOMA-IR, Homeostasis Model Assessment insulin resistance index; CIR, corrected insulin response
*Time points (min) during an oral glucose tolerance test, **Calculated as a reduced Matsuda index using data for 0', 30', 120' min during OGTT.

Supplementary Fig. 4: **Glucose and C-peptide levels during OGTT in *RFX6* heterozygotes**.

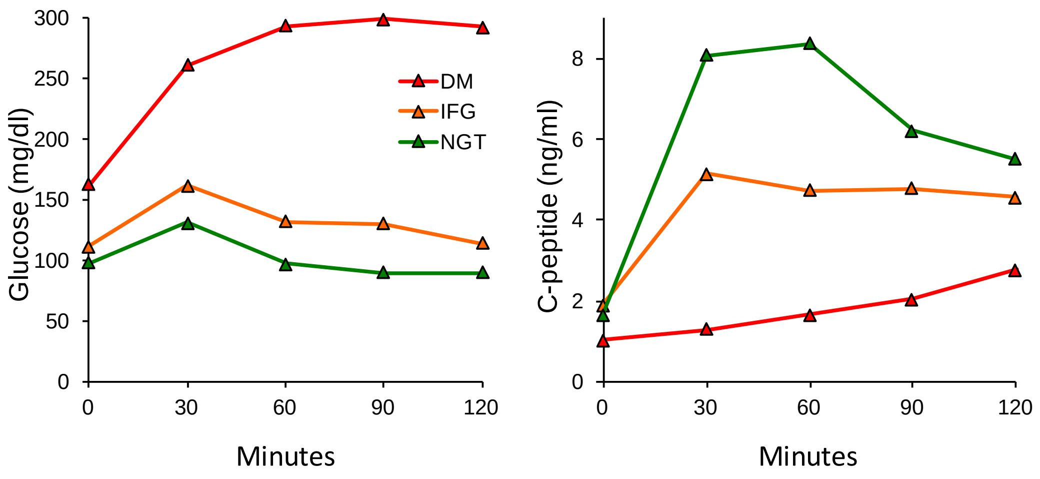

NGT=normal glucose tolerance (green, n=5-6, for C-peptide and glucose levels, respectively), IFG= impaired fasting glucose (orange, n=4) DM= overt diabetes (red, n=1-3). Data are mean values from 13 non-Finnish and Finnish *RFX6* heterozygotes, 5 Male/8 Female, mean age 42 years and BMI 26 kg/m^2.
